# White Blood Cell Count in Elderly Is Clinically Useful in Predicting Long-Term Survival

**DOI:** 10.1155/2014/475093

**Published:** 2014-01-29

**Authors:** Göran Nilsson, Pär Hedberg, John Öhrvik

**Affiliations:** ^1^Centre for Clinical Research, Västmanland County Hospital, Uppsala University, 721 89 Västerås, Sweden; ^2^Department of Clinical Physiology, Västmanland County Hospital, 721 89 Västerås, Sweden; ^3^Department of Medicine, Karolinska Institutet, 171 76 Stockholm, Sweden

## Abstract

*Introduction*. White blood cell (WBC) count is often included in routine clinical checkups. We determined the prognostic impact of WBC count on all-cause, cardiovascular, and noncardiovascular mortality during an 11-year followup in a general population of 75-year-olds. *Study Population*. The study included 207 men and 220 women comprising 69% of the invited 75-year-olds in a defined geographical area. *Main Results*. The median WBC count (in 10^9^/L) was 6.3 (interquartile range 5.4–7.2) for men and 5.7 (4.9–6.8) for women, *P* < 0.001 for sex difference. The hazard ratio (HR) for all-cause mortality per 10^9^/L increase in WBCs was 1.16 (95% confidence interval, 1.03–1.32; *P* = 0.016) in men and 1.28 (1.10–1.50; *P* = 0.002) in women. These HRs were essentially unchanged by adjustment for established risk factors (current smoking, known hypertension, prior myocardial infarction, known diabetes, total cholesterol, high-density lipoprotein cholesterol, and body mass index). Furthermore, increased WBC count was significantly associated with cardiovascular mortality in both sexes and with noncardiovascular mortality in women. *Conclusions*. The WBC count deserves attention as a potentially clinical useful predictor of survival in the 75-year-olds, especially among women.

## 1. Introduction

The white blood cell (WBC) count is marker of systemic inflammation. It is determined routinely by means of well-standardized automated methods at low cost and with high precision. Consequently, the WBC count is often included in routine clinical checkups. Data from multiple observational studies have demonstrated that WBC count has an independent ability to predict all-cause mortality [1–4], cancer mortality [5, 6] and cardiovascular diseases and mortality [7]. Reports [2, 8, 9] suggest that the predictive ability of the WBC counts also applies to the elderly but there is a need for further studies on this topic, particularly among elderly women.

The aim of the present study was to determine the prognostic impact of WBC count on all-cause, cardiovascular, and noncardiovascular mortality during an 11-year followup in a general population of 75-year-olds.

## 2. Methods 

### 2.1. Study Population

The city of Västerås (130,000 inhabitants) situated in central Sweden has a population that is socioeconomically representative for the country. In 1997 a random sample of 618 of the 1,100 inhabitants born in 1922 (i.e., 75 year old) were invited to participate in a cardiovascular health survey. Four hundred thirty-two individuals (210 men and 222 women) finally accepted the invitation, corresponding to a participation rate of 70%. Reasons for nonparticipation were: Unknown (*n* = 46), never reached (*n* = 29), unwilling due to diseases under treatment (*n* = 54), locomotive impairment (*n* = 28), language difficulties or logistical problems (*n* = 26), or died before examination (*n* = 2). Due to missing values in WBC counts (4 subjects), the examined cohort finally comprised 208 men and 220 women (69% of invited individuals).

The study was approved by the research ethics committee of Uppsala University, Sweden, and was conducted in accordance with the Declaration of Helsinki. All subjects gave their informed consent.

### 2.2. Blood Analyses

The blood samples were collected in a fasting state in the morning. WBCs were counted with an automated blood cell counter, Cell-Dyn 3500 (Abbott). Serum triglycerides, total cholesterol, and high-density lipoprotein cholesterol (HDL-C) were determined enzymatically on an automated analyser system (Hitachi 717, Boehringer Mannheim). Low-density lipoprotein cholesterol (LDL-C) was calculated using the Friedewald formula [10]. The blood glucose samples were treated with a haemolytic reagent (Merck Diagnostica) and glucose was determined enzymatically with glucose dehydrogenase on a Cobas Mira analyser. Plasma glucose was computed from venous whole blood glucose using the formula: plasma glucose = 0.558 + 1.119* whole blood glucose [11].

Blood pressure (BP) was measured to the nearest five mm Hg with a mercury sphygmomanometer with the subjects in a supine position having rested for five minutes.

### 2.3. Prospective Followup

All-cause mortality served as the primary end point. By means of the Swedish population register, the study cohort was followed for all-cause mortality from the index examination in 1997 until November 1, 2008. Only one individual (a man) was lost to followup (reason: migration). Causes of death until November 1, 2008, were obtained from the Swedish Cause of Death Register. The 10th revision of the International Statistical Classification of Diseases (ICD) was used to identify causes of death. For the present analyses, causes of death were grouped into two categories: cardiovascular, ICD 10, I00-I99, or noncardiovascular comprising all other causes of death. The study was not powered for analyzing more detailed categories of death causes.

### 2.4. Statistical Analyses

Continuous variables were summarized by medians and interquartile ranges and categorical variables by numbers and proportions. The Wilcoxon-Mann-Whitney rank sum test was used to compare continuous variables and Fisher's exact test to compare categorical variables.

Crude and adjusted prospective associations of the WBC count with mortality were assessed by hazard ratios (HR) and corresponding 95% confidence intervals (CI) using uni- and multivariable Cox proportional hazard regression. For continuous variables, the assumption of proportional hazards (PH) was assessed by examining the variables' interaction with time or a function of time (e.g., log(time)) in a Cox model. The PH assumption for categorical variables was assessed by visual inspection of the log (−log(cumulative survival)). Cumulative survival was estimated by means of the Kaplan-Meier survival curves. Associations between continuous variables were assessed by Spearman's rank correlation. The sex disparity in the strength of the association between WBC count and other markers was assessed by including the interaction term between sex and WBC count in a regression model with the marker as dependent variable.

A two-sided *P* value <0.05 was regarded as statistically significant in all analyses. IBM SPSS version 20 was used for the analyses.

## 3. Results

The distribution of the WBC counts among the participants at baseline was positively skewed. The ranges of the WBC counts (in 10^9^/L) were 3.0–12.0 for men and 2.9–10.6 for women. The median (interquartile range) was 6.3 (5.4–7.2) for men and 5.7 (4.9–6.8) for women. The mean (SD) was 6.36 (1.44) for men and 5.90 (1.46) for women. The sex disparity was statistically significant (*P* < 0.001).

Table 1 shows the baseline characteristics for men and women stratified by survival status. Of note only WBC count differed significantly between survivors and nonsurvivors in both sexes (higher in nonsurvivors); in addition known hypertension, prior myocardial infarction, and lower HDL-C levels were significantly more common in nonsurviving men and higher plasma glucose levels in nonsurviving women.

There were strong correlations between WBC count and plasma glucose and HDL-C, respectively, in women (Spearman correlation 0.29; *P* < 0.001 and Spearman correlation −0.25; *P* < 0.001) but not in men (Spearman correlation 0.09; *P* = 0.22 and Spearman correlation 0.01; *P* = 0.88). The sex disparity in the strength of these associations was significant (*P* = 0.030 and *P* < 0.001).

During a median followup of 11.4 years (range 0.2–11.8 years) 108 (52%) men and 67 (30%) women died. The total number of person-years with followup was 4088 implying 4.3 deaths per 100 person-years at risk (men 5.9; women 3.0). In men there were 48 deaths from cardiovascular causes and 60 from noncardiovascular causes (including 31 attributed to malignancy). The corresponding figures for women were 32 deaths from cardiovascular causes and 35 from noncardiovascular causes (including 13 attributed to malignancy). The cause of death was unknown in one man and three women.

The mortality among the 30% of invited individuals who did not participate in the investigation was considerably higher (67%) than among the participants.

Table 2 shows the Cox regression analyses of WBC count and all-cause, cardiovascular, and noncardiovascular mortality stratified by sex. Three different models were assessed: model 1 unadjusted HR for WBC count, model 2 HR for WBC count adjusted for current smoking and known hypertension, and model 3 HR for WBC count adjusted for current smoking, known hypertension, prior myocardial infarction, known diabetes, total cholesterol, HDL-C, and BMI. Model 2, which adjusts for the two most significant risk factors, was used for the cardiovascular and noncardiovascular mortality since the relatively few events in these categories did not allow further adjustments. The prognostic ability of the WBC count for all-cause mortality was considerably better in women than in men, but the sex disparity did not reach statistical significance (*P* = 0.34).

In an additional analysis we excluded deaths within 5 years, thereby reasonably excluding prevalent severe disease at the time of inclusion in the study. For all-cause mortality, the HR (95%CI) was 1.11 (0.96–1.29; *P* = 0.175) in men and 1.27 (1.08–1.51; *P* = 0.004) in women.

**Figure 1:**
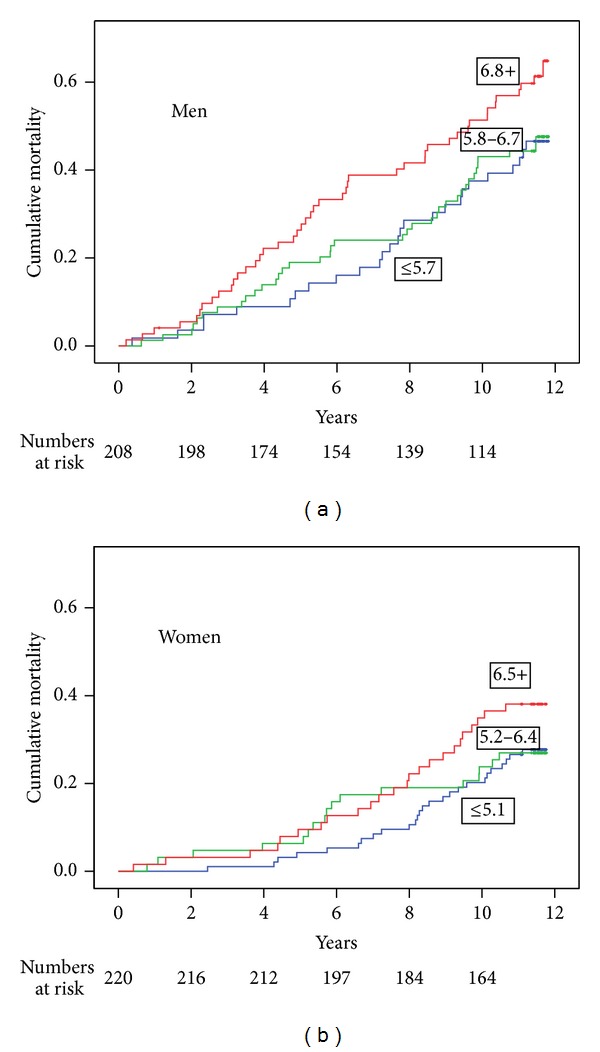
Kaplan-Meier curves showing cumulative all-cause mortality according to sex-specific WBC count tertiles.

Figure 1 shows the Kaplan-Meier curves for all-cause mortality according to sex-specific WBC count tertiles. Of note women with a WBC count in the two lowest tertiles (≤6.4 × 10^9^/L) had a considerably lower cumulative mortality during followup (27%) compared with the mortality among men (62%) in the highest tertile (WBC count ≥6.8 × 10^9^/L).

## 4. Discussion

### 4.1. Principal Finding

The principal finding in the present community-based study of 75-year-olds is a considerable long-term prognostic significance of the basic WBC count, especially among women. Determination of the WBC count is a well standardized and cost-effective procedure with excellent precision and it is often included in routine checkups. Thus, the present finding is of potential clinical importance. We previously reported the poor prognostic value of serum cholesterol in the present population [12]. Evidently, the WBC count has a much stronger prognostic ability with regard to total mortality and cardiovascular mortality than total cholesterol and LDL-C among the elderly.

### 4.2. Strengths and Limitations

The restriction of the present investigation to one age class made it possible to leave age out of account as covariate, thereby enabling us to draw conclusions about WBC counts and mortality despite a relatively low number of participants in the study. This advantage, of course, was obtained at the cost of generalizing our conclusions to people not 75 years of age and to people living in other geographical areas. However, our conclusions seem reasonably applicable to north Europeans and white North Americans in their seventies. The strength of the present study is increased by a relatively long time of followup.

The examined communitybased cohort was well defined and the participation rate was rather high. Reasons for nonparticipation were accounted for and included a substantial fraction of individuals already under medical attention. This is a likely explanation for the higher mortality among nonparticipants than among participants. Similar studies from other centers need to confirm the results from the present study.

### 4.3. Comparison with Other Studies

It is well known that the WBC count is higher among men than among women [3, 13]. The reason for this sex disparity is unknown. Modification of inflammatory reaction owing to the necessity of female acceptance of the genetically different fetal tissue during pregnancy may be one speculative reason for the sex disparity.

Multiple observational studies have demonstrated that WBC counts have substantial ability to predict all-cause mortality [1–4, 14], cardiovascular mortality [14, 15], cerebrovascular mortality [7], and cancer mortality [5, 6, 14] as well as incident coronary heart diseases [15, 16]. Only a few of these studies have included women [3, 6, 15]. In these observational studies broad age categories of mainly middle-aged people have been evaluated as homogenous groups with no opportunity to distinguish prognoses for distinct age classes, such as the 75-year-olds of the present study.

The sex difference in WBC count found in the present study corresponds to that found in people over 75 years of age in the United States National Health and Nutrition Examination Survey (NHANES) [17]. Furthermore the NHANES study demonstrated a mean WBC count slightly on the rise in those above 65 years of age [17].

Each age class represents survivors from younger age classes. Consequently, clinically established risk factors among middle-aged people may not be applicable to elderly people, whereas other factors, exemplified by the WBC count, may assume greater importance. Our finding of this test's relatively strong prognostic ability among the elderly is clinically important.

Concerning the prognostic aspect of the WBC count, two past studies have included elderly women. In a 10-year followup of 705 men and women, more than 85 years old, in Leiden in The Netherlands, Willems et al. [8] found results similar to ours. However, their analyses were not stratified by sex. Margolis et al. [15] reported a followup of 72,000 women from the Women's Health Initiative Observational Study. Higher WBC counts predicted cardiovascular events and total mortality during a mean followup of 6.1 years also after multifactor adjustment including age. The oldest group (70–79 years) comprised 22% of the cohort, but no separate analyses were performed for this age group. Women in the top quartile (≥6.7 × 10^9^, cells/L) had 50% higher risk for total mortality than women in the lowest quartile (≤4.7 × 10^9^, cells/L).

Concerning elderly men, Weijenberg et al. [2] reported 5-year survival among 884 randomly selected men, aged 65–84 years, from the general population. The excess mortality risk with increasing WBC count was similar to our results. Notably, the followup time for Weijenberg et al. [2] was considerably shorter than that of the present study (5 versus 11 years). In the present study we observed a significantly higher risk of mortality among women even if deaths within 5 years were excluded.

### 4.4. Possible Mechanisms

The WBC count tends to cluster with other established risk factors such as tobacco smoking [14, 18] as well as HDL-cholesterol and triglycerides [9, 13]. However, the risk excess found in the present analyses was practically unchanged after adjustment for these factors.

The pathophysiological mechanisms that link elevated WBC count to increased mortality are not well understood. Thus, it is not known whether elevated WBC count is involved directly in the pathogenesis of vascular diseases or is merely a risk indicator of other factors causing vascular damage [19]. The same uncertainty applies also to the well-known relation between elevated WBC count and diabetes [20]. There is clear evidence that neutrophil is the subgroup of WBC that is most strongly associated with coronary risk [16]. This finding might provide clues as to the association between WBC counts and cardiovascular death.

### 4.5. Clinical Implications

By means of our data, the mortality risk among elderly people at different levels of basal WBC counts (Figure 1) can be estimated in a cost-effective manner during clinical checkups.

Of note, this prognostic ability contrasts with the serum cholesterol's lack of ability to predict all-cause mortality among the elderly [12]. The within-subject variability compared with the between-subject variability is similar to WBC count and serum cholesterol [21]. Thus, determination of the WBC count seems more useful than the determination of the serum cholesterol in a clinical geriatric context.

## Figures and Tables

**Table 1:** Sex-specific baseline characteristics of the study cohort according to survival status. Categorical variables are shown as number (%) and continuous variables as median (interquartile range).

	Survivors		Nonsurvivors	
	Men (*n* = 99)	Women (*n* = 153)	Men (*n* = 108)	Women (*n* = 67)
Current smoker	9 (9)	8 (5)	18 (17)	8 (12)
Known hypertension	14 (14)^†^	39 (25)	41 (38)^†^	26 (39)
Known diabetes	6 (6)	9 (6)	9 (8)	8 (12)
Prior myocardial infarction	9 (9)^†^	4 (3)	22 (20)^†^	6 (9)
Statin medication	1 (1)	4 (3)	6 (6)	3 (4)
WBC count (10^9^/L)	6.1 (5.4–6.8)^†^	5.6 (4.7–6.7)^‡^	6.4 (5.5–7.4)^†^	5.8 (5.1–7.1)^‡^
BMI (kg/m^2^)	25.4 (23.1–26.8)	26.2 (23.7–29.3)	25.1 (23.7–27.3)	25.8 (23.4–28.3)
Plasma glucose (mmol/L)	5.8 (5.4–6.4)	5.9 (5.4–6.4)^‡^	5.9 (5.4–6.6)	6.0 (5.5–7.2)^‡^
Systolic BP (mmHg)	160 (144–180)	165 (150–190)	160 (150–180)	165 (150–180)
Diastolic BP (mmHg)	83 (75–90)	85 (80–90)	85 (80–91)	85 (75–90)
Total cholesterol (mmol/L)	6.0 (5.4–6.8)	6.6 (5.8–7.3)	5.9 (5.3–6.5)	6.5 (5.9–7.0)
LDL-cholesterol (mmol/L)	3.8 (3.2–4.7)	4.1 (3.4–4.8)	3.8 (2.9–4.3)	4.2 (3.6–4.8)
HDL-cholesterol (mmol/L)	1.4 (1.2–1.7)^†^	1.6 (1.4–2.0)	1.4 (1.2–1.5)^†^	1.5 (1.2–1.9)
Triglycerides (mmol/L)	1.4 (1.0–1.8)	1.4 (1.1–2.1)	1.6 (1.1–2.0)	1.5 (1.1–2.0)

^†^Significant difference between survivors and nonsurvivors in men.

^‡^Significant difference between survivors and nonsurvivors in women.

**Table 2:** Cox regression analyses of all-cause, cardiovascular, and noncardiovascular mortality according to WBC count stratified by sex (*n* = 207 men and  *n* = 220 women).

	HR	95% CI	*P* value
Men			
All-cause mortality			
Model 1	1.16	1.03–1.32	0.016
Model 3	1.12	0.98–1.28	0.092
Cardiovascular mortality			
Model 1	1.21	1.01–1.45	0.038
Model 2	1.13	0.95–1.35	0.181
Noncardiovascular mortality			
Model 1	1.12	0.95–1.33	0.171
Model 2	1.09	0.92–1.30	0.331
Women			
All-cause mortality			
Model 1	1.28	1.10–1.50	0.002
Model 3	1.23	1.03–1.47	0.020
Cardiovascular mortality			
Model 1	1.30	1.04–1.62	0.021
Model 2	1.21	0.96–1.53	0.110
Noncardiovascular mortality			
Model 1	1.27	1.03–1.58	0.028
Model 2	1.20	0.95–1.52	0.119

All hazard ratios (HR) are per 10^9^/L increase in WBC count. Model 1: crude HR, model 2: HR adjusted for current smoking and known hypertension, and model 3: HR adjusted for current smoking, known hypertension, prior myocardial infarction, known diabetes, total cholesterol, HDL-cholesterol, and BMI.
